# Multiple extraarticular giant cell tumors

**DOI:** 10.1016/j.jdcr.2024.04.036

**Published:** 2024-05-08

**Authors:** Faraz Yousefian, Marianne Cortes, Benjamin Kahn, Marcus Goodman, Nicole Kounalakis

**Affiliations:** a Department of Dermatology and Mohs Surgery, Philadelphia College of Osteopathic Medicine, Roswell, Georgia; b Goodman Dermatology, Roswell, Georgia; c Nova Southeastern University Kiran C. Patel College of Osteopathic Medicine, Davie, Florida; d Northside Hospital Cancer Institute Melanoma and Sarcoma Specialists of Georgia, Atlanta, Georgia

**Keywords:** extraarticular, oncology, soft tissue tumor, surgery, tenosynovial giant cell tumors

## Introduction

Tenosynovial giant cell tumors (TGCTs) are uncommon, indolent, painless, and benign lesions of soft tissue. They have a higher incidence in women between the ages of 30 and 50 years old.^1^ TGCT commonly arises from a tendon sheath or synovial lining adjacent to small joints and are classified based on their growth pattern (localized vs diffuse) and location (intraarticular vs extraarticular).^2^ Despite their benign nature, these tumors can cause extensive local destruction with recurrence rates after excision being as high as 55%.^3^ Although they can occur in any joint, >75% of all cases occur intraarticularly, making the presence of extraarticular extensions uncommon and frequently misdiagnosed.^4^^,^^5^ Extraarticular presence of TGCT affects the synovial lining, demonstrating a more extensive growth pattern requiring a more intricate treatment plan. The purpose of our report is to discuss the surgical complexity of the extraarticular manifestation of the localized-subtype of TGCT (lTGCT) through the case of a patient presenting with the disease.

## Case presentation

A 51-year-old woman with no significant past medical history presented with 2 asymptomatic firm soft tissue nodules on her back that had grown slowly over the past year. She denied systemic symptoms in addition to any recent trauma, traveling, or sick contacts. Her family did not have a history of similar lesions or malignancy.

**Fig 1:**
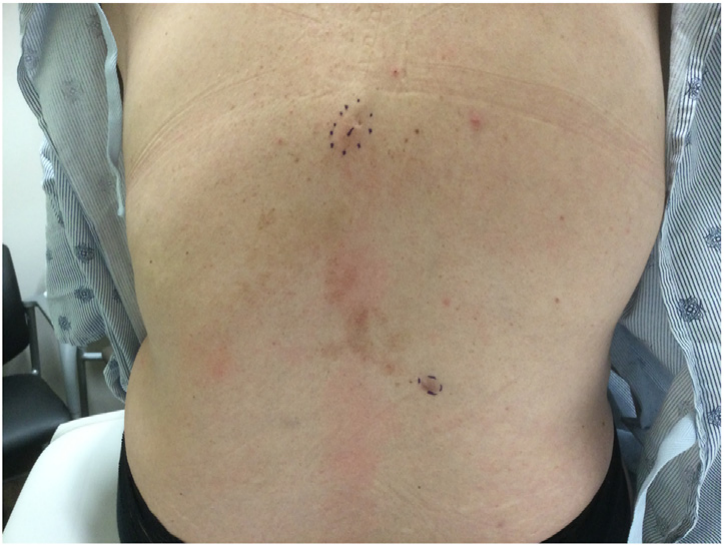
Physical examination revealing 1 cm upper portion of the left mid back mass and 1.5 cm on the lower portion of the right back mass.

**Fig 2:**
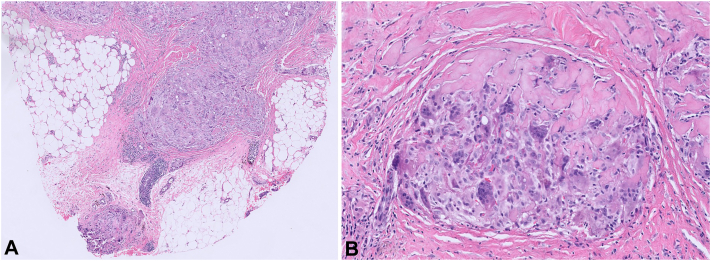
Histopathology revealing sheet-like proliferation of numerous osteoclast-like multinucleated giant cells admixed with uniform spindle cells within the dermis between collagen bundles. (Original magnifications: **A,** ×10; **B,** ×40.)

**Fig 3:**
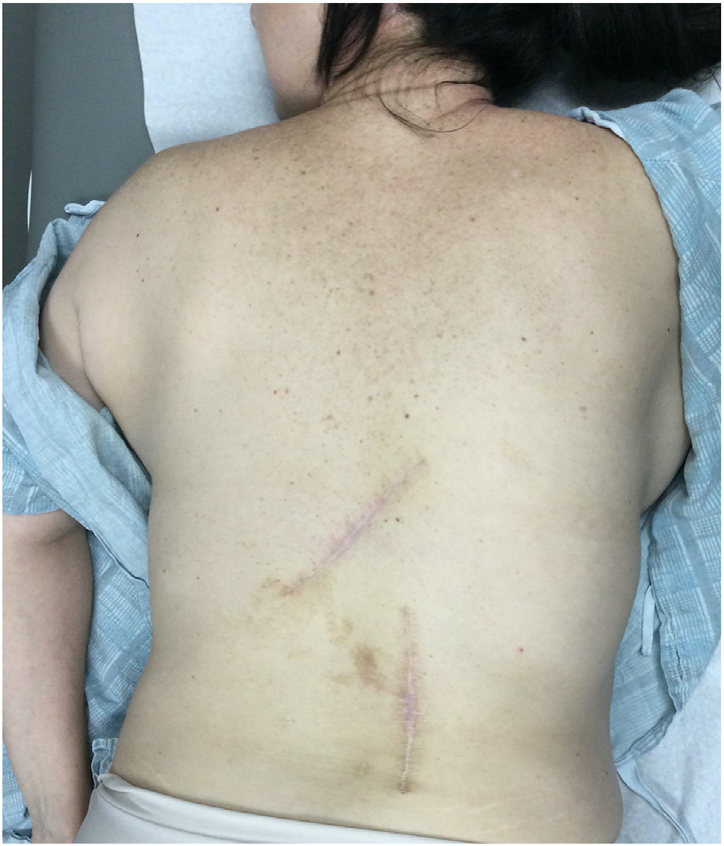
Postsurgical scars after the second reexcision of both lesions with clear margins.

Examination revealed 2 masses on the left and right mid back measuring 1 and 1.5 cm, respectively (Fig 1). Punch biopsy of both lesions revealed sheet-like proliferation of numerous osteoclast-like multinucleated giant cells admixed with uniform spindle cells within the dermis between collagen bundles (Fig 2). Clinical and histologic findings were consistent with giant cell tumors of the soft tissue without any tendinous involvement. Following discussion of treatment options including observation, wide local excision, and Mohs micrographic surgery, the patient opted for surgical excision of both lesions with 1 cm margins. Surgical pathology confirmed 0.8 cm residual disease with clear margins in the lower portion of the right back but 1.8 cm tumor with diffusely positive margins in the upper portion of the left mid back. The patient requested the complete removal of the positive margins and the subsequent reexcision of the upper portion of the left mid back tumor with an additional 1 cm margins led to clear margins (Fig 3). Three months after her reexcision, 2 additional TGCTs developed: a 1.7 cm mass in her upper portion of the right groin skin and a 3 cm mass in her right inferior buttock skin. Both were cleared with 1 cm margins. The patient requested medical oncology referral given the number and frequency of the tumors. Staging workup did not show any metastatic spread. She was referred to a genetic counselor, who did not offer any additional evaluation for a germline mutation or identify any obvious link to a genetic syndrome. She has been followed now for 6 months without any new disease.

## Discussion

lTGCT is distinguished as a locally benign tumor commonly affecting focal portions of the synovium within the small joints of the lower extremity in its extraarticular subtype.^3^^,^^4^ The incidence range for lTGCT was estimated to be 30.3 to 39 per million person-years, and despite the rarity of these tumors, TGCT continues to be one of the most common soft tissue tumors of the foot and ankle region.^4^ lTGCT can cause localized joint destruction along with debilitating symptoms, there have also been a few documented cases of malignant transformation in the literature.^5^ The overall pathogenesis has yet to be understood, however, it has been hypothesized that chromosomal translocations involving chromosome 1p13 lead to an overexpression of macrophage colony stimulating factor, triggering an accumulation of macrophages to form a tumor-like mass.^2^^,^^6^ A delay in diagnosis is exacerbated by the uncommon nature of the tumor, vague symptomatology, and a lack of standardized diagnosis criteria.

Diagnostic imaging often involves conventional radiographs and magnetic resonance imaging. Advanced disease is characterized by tissue swelling, loss of joint space and periarticular erosion of bone being synonymous.^2^ A distinguishing “blooming effect” on magnetic resonance imaging, marked by accentuated low signal intensity due to hemosiderin is distinctive.^7^ The differentiation between extraarticular and intraarticular can also be made via magnetic resonance imaging, with extraarticular tumors showing a multinodular appearance in contrast to the villous pattern present in intraarticular subtypes.^4^ A diagnosis can be further confirmed via histologic examination depicting osteoclast-like multinucleated giant cells, hemosiderin pigments, and spindle-shaped mononuclear cells.^1^^,^^8^

Without a standard set of guidelines for treatment, options can include surgical resection, external beam radiotherapy, cryotherapy, arthroplasty, or amputation.^3^ Although surgical resection continues to be the predominant treatment modality, the invasive nature of the procedure and high recurrence rates following resection have led to adjuvant or multimodality treatments such as radiotherapy, radiation synovectomy, radioactive colloid, or novel colony stimulating factor 1 receptor inhibitors to be used for advanced disease.^2^^,^^6^

Due to the limited extent of the localized-subtype, an arthroscopic surgical resection is often recommended. An open surgical approach would be better suited if an extraarticular component is additionally present to ensure complete removal.^5^ A diffuse subtype creates more of a challenge due to the increased likelihood of spreading pathologic tissue, due to this, adjuvant therapies in combination with resection are generally recommended.^5^^,^^6^ Careful monitoring with imaging after surgery with continuous follow-up to ensure complete removal should be considered.

In our case, the patient opted for monitoring despite the presence of continued positive margins status postsurgical excision due to her anxiety, stemming from difficulties in clearing the mass following a previous unsuccessful attempt. In planning for future management, we explored multiple options including additional surgeries, larger excisional margins, and utilizing imaging to verify complete removal. Irrespective of this challenge, the treatment plan should be tailored to meet the specific needs of the individual patient.

Although extraarticular lTGCT is a benign soft tissue tumor, the consequences of delayed treatment can lead to detrimental functional impairment. Managing this tumor requires considering its growth pattern, location, and individual patient’s preferences. Given its intricacy, particularly in the extraarticular form, a multidisciplinary approach and vigilant postoperative monitoring are crucial in ensuring complete removal and sustained remission. Further studies would help develop a standardized treatment protocol to minimize recurrence and increase the quality of life in affected patients.

## Conflicts of interest

None disclosed.
